# Thyroid Metastasis from Primary Breast Cancer

**DOI:** 10.3390/jcm12072709

**Published:** 2023-04-04

**Authors:** Armando Patrizio, Silvia Martina Ferrari, Giulio Stoppini, Elena Palmisano, Giusy Elia, Francesca Ragusa, Sabrina Rosaria Paparo, Eugenia Balestri, Valeria Mazzi, Chiara Botrini, Agnese Proietti, Fausto Famà, Salvatore Benvenga, Alessandro Antonelli, Poupak Fallahi

**Affiliations:** 1 Department of Emergency Medicine, Azienda Ospedaliero-Universitaria Pisana, 56124 Pisa, Italy; 2 Department of Clinical and Experimental Medicine, University of Pisa, 56126 Pisa, Italy; 3 Department of Surgical, Medical and Molecular Pathology and Critical Area, University of Pisa, 56126 Pisa, Italy; 4 Department of Human Pathology in Adulthood and Childhood “G. Barresi”, University Hospital “G. Martino”, 98125 Messina, Italy; 5 Department of Clinical and Experimental Medicine, University of Messina, 98125 Messina, Italy; 6 Master Program on Childhood, Adolescent and Women’s Endocrine Health, University of Messina, 98125 Messina, Italy; 7 Interdepartmental Program of Molecular and Clinical Endocrinology and Women’s Endocrine Health, Azienda Ospedaliera Universitaria Policlinico “G. Martino”, 98125 Messina, Italy; 8 Department of Translational Research and New Technologies in Medicine and Surgery, University of Pisa, 56126 Pisa, Italy

**Keywords:** breast cancer, thyroid metastasis, thyroid ultrasound, secondary thyroid cancer

## Abstract

Breast cancer (BC), the most commonly diagnosed malignancy, frequently metastasizes to the bone, lungs, brain and liver at advanced stages, whereas the thyroid gland represents a rare target site for secondary disease. We examined the most recent literature about thyroid metastasis (TM) from BC after we encountered a peculiar case of a 71-year-old woman who developed sudden dysphagia, severe hypothyroidism and hypoparathyroidism due to TM 18 years after the diagnosis of her primary cancer. Based on published data, the prevalence of TM in BC ranges from 3% to 34%, with a median onset time of 48.2 months, although longer time intervals are not infrequent. TM negatively impacts the prognosis of these patients, however thyroid surgery can limit the local disease burden. Therefore, we suggest that clinicians involved in the follow-up care of BC patients should consider a differential diagnosis of secondary thyroid malignancy when incidental lesions are diagnosed during radiological evaluations or local symptoms affect the cervical region, even many years after the diagnosis of the primary cancer.

## 1. Introduction

Breast cancer (BC) remains the most commonly diagnosed malignancy and the second highest cause of cancer-related mortality worldwide [1]. Bones and visceral organs, such as the lungs, brain and liver, are common target sites for metastatic BC [2]. Although the thyroid has one of the highest blood supplies (4 to 6 mL/min/g) [3], metastatic carcinoma to the thyroid is rare, and it is generally derived from primary tumors of the kidney, gastrointestinal system, lungs, skin, and, rarely, breast [4]. The presence of thyroid metastasis (TM) usually implicates a poor prognosis [5,6]. However, the improvement of diagnostic methods and cancer therapies, and the subsequent improvement in terms of the survival rate of patients, has led to a rise in the incidence of thyroid metastatic cancers [4]. Here, we report a rare case of a patient with breast cancer metastasis to the thyroid gland which became overt due to severe dysphagia, and symptomatic hypothyroidism and hypocalcemia 18 years after the primary cancer diagnosis. Moreover, to improve the management of this uncommon but potentially lethal diagnosis, we review the evidence regarding the clinical diagnosis and treatment of TM from BC.

## 2. Methods

We performed a comprehensive review of the English-language literature published between January, 2000 and the end of January, 2023, extracted from the PubMed/Medline database, on studies about patients who developed TM after a diagnosis of BC. We included original articles, reviews, opinion pieces, commentaries, case series and case reports. The search terms, used both separately and in combination, included: “thyroid metastasis”, “secondary thyroid cancer”, “secondary thyroid malignancy”, “breast cancer”, and “breast malignancy.” Papers focused on metastasis of thyroid cancer and articles published in languages other than English were excluded.

## 3. Our Experience

A 71-year-old lady came to our attention due to fatigue and worsening dysphagia, first to solids and then also to liquids. She had a history of left BC, diagnosed 18 years prior and treated with a quadrantectomy and axillary lymphadenectomy, followed by chemotherapy (Adriamycin 60 mg/mq and Cyclophosphamide 600 mg/mq for four cycles), radiotherapy and hormonal therapy (Tamoxifen followed by Anastrozole for five years). At the time, the histopathology disclosed a 2 cm invasive ductal carcinoma, moderately differentiated (G2) with concomitant homolateral axillary lymph nodes metastasis (T1N1M0). Her past medical history also included hypertension, osteopenia and osteoarthritis with a Baker’s cyst in the right knee.

During the work-up for dysphagia, high TSH values were found (TSH 70 mUI/L, normal range 0.27–4.20 mUI/L), while the patient’s results indicated euthyroid during previous laboratory investigations (at least three measurements were taken in the year prior and were found to be within the normal limits, see Table 1). The family history did not reveal any noteworthy thyroid pathologies. The physical examination of the neck showed the presence of palpable nontender bilateral thyroid nodules. The brain MRI excluded the neurologic etiology of dysphagia, while a contrast esophagram documented a hypertonic dyskinesia at the cervical–dorsal passage, with the absence of intrinsic organic lesions. At our department, the repeated thyroid function test (TSH 27.7 mUI/L [0.4–4], fT3 2.55 ng/L [2.5–70.70], fT4 0,92 ng/dL [0.70–1.70]) confirmed overt hypothyroidism; the levels of thyroglobulin, calcitonin and anti-thyroglobulin antibodies were normal, however there was a weak positive result for anti-thyroperoxidase antibodies (35 UI/mL, range of normal up to 10). We also detected low calcium levels (7.2 mg/dL, [8.6–10.2]) with inappropriate parathyroid hormone (PTH) levels in the lower-end of normal range (12 ng/L, [8–40]), consistent with primary hypoparathyroidism.

**Figure 1:**
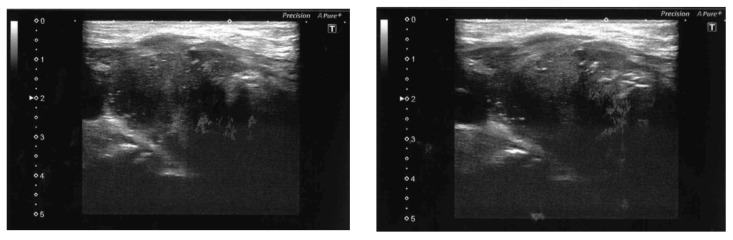
Heterogeneous appearance with diffuse microcalcifications on ultrasonography of the left thyroid lobe.

An ultrasound of the neck was then performed which found a multinodular, but normal-sized, thyroid gland. In particular, a hypoechoic right midlobar nodule, inhomogeneous with multiple calcifications of 27 × 18 × 16 mm, and a left isoechoic inhomogeneous midlobar nodule with multiple small calcifications of 21 × 9 ×11 mm (Figure 1) were reported. The vascularization of the thyroid parenchyma by power Doppler ultrasonography was not significantly altered, and the nodules showed perinodular vascularization. Finally, no cervical lymphadenopathy was detected.

Oral liquid levothyroxine was started at a dose of 25 mcg daily, and the Fine needle aspiration cytology (FNAC) of the larger nodule revealed cytological findings compatible with a poorly differentiated malignancy (TIR5, Italian consensus for the classification and reporting of thyroid cytology, [7]). For hypocalcemia and hypovitaminosis D, therapy with calcium carbonate, calcitriol and cholecalciferol was introduced.

**Figure 2:**
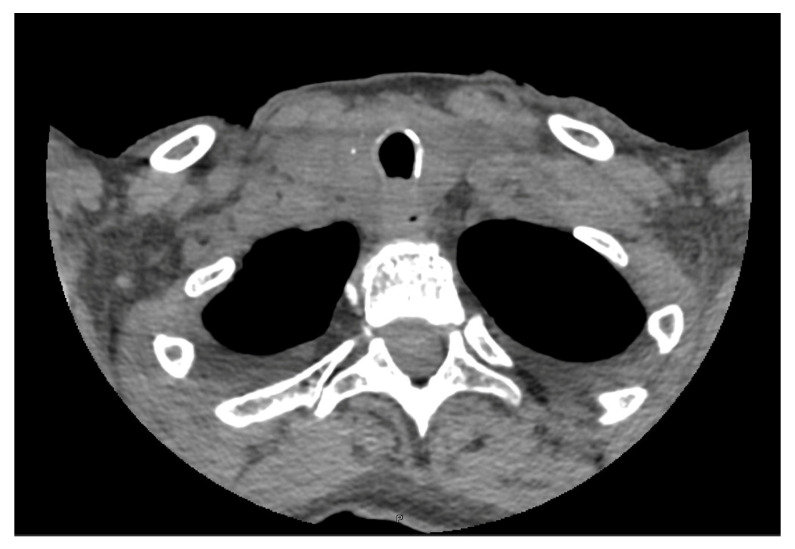
CT of the neck demonstrating narrowing of the cervical portion of the esophagus by TM.

The patient was then admitted to our hospital due to the further deterioration of the dysphagia and her general condition. She was submitted to a whole-body computed tomography (CT) that confirmed the extrinsic compression of the esophagus by a cervical mass located at the thyroid bed (Figure 2), sub-clavicular and paratracheal lymphadenopathy, a hepatic focal centimeter lesion at the fourth segment, abdominopelvic effusion and diffuse bone involvement (dorsal spine, bilateral hip). One month after our first visit, she was subjected to a partial thyroidectomy with significant symptom relief. The pathology of the thyroid samples disclosed a secondary malignancy of poorly differentiated breast cancer with immunophenotypes CK7+, CK 20-, GATA3+, TTF-1-, P40-, S100-, CDX-2-, Thyroglobulin-, Pax8-, and P63-. As per the specific request of the oncologist, the biological parameters of the expression of the hormone receptors (HR) were searched, which found: estrogen receptor (ER) 90% positive, progesterone receptor (PgR) 10%, Ki-67 30% and human epidermal growth factor receptor 2 (HER2) score 1+, which is considered negative.

Subsequently, anticancer therapy was started, consisting of 10 cycles of chemotherapy with Paclitaxel (80 mg/mq). The patient responded to it with a return to oral enteral nutrition, weight recovery and a substantial general clinical benefit. Furthermore, thyroid function was restored following the titration of the replacement therapy with levothyroxine, up to 100 mcg per day (Table 1), and blood calcium levels fell to within normal limits, for which the patient is currently taking 25,000 UI of cholecalciferol per month and 1 mcg per day of calcitriol. At the last CT-scan follow-up, which took place a year after the surgery, there was a significant improvement in the disappearance of the hepatic lesion and the stability of other metastases. The patient is currently alive and no further endocrine impairment has emerged.

## 4. Current Knowledge

Secondary malignancy of the thyroid is a rare event. To define the real rate of TM is challenging; in autopsy studies, it ranges from 1.25% to 24% [8]. In previous studies, BC has been described as a variable primary source of TM [9,10], with a prevalence ranging from 3% to 34% of all thyroid metastases [11]. A recent review estimated the frequency of BC-related TM to be 7.8%, while for renal cell carcinoma (RCC) it is 48% [12]. Currently, the etiology of thyroid metastasis from primary carcinoma is not clear. In fact, although the thyroid gland is highly vascularized, it does not represent a frequent target for systemic distant disease. One study speculated that pre-existing thyroid diseases (i.e., thyroiditis and goiter) could facilitate the initiation of TM originating from cancers of different tissues, changing the thyroid microenvironment levels of oxygen and iodine [5]. On the other hand, a previous study has shown that different BC subtypes display strong associations with site-specific metastasis target tissues [13]. Moreover, BC and differentiated thyroid cancer (DTC) are the most common malignancies among women and endocrine cancers, respectively, and their simultaneous presentation in the same patient should raise the suspicion of synchronous primary cancers, especially in light of the shared risk factors for these two types of tumors. [14,15,16]. Rare cases of TM from BC within another primary malignant thyroid cancer (“tumor-to-tumor metastasis”) have also been reported [17,18]. Previous experiences reported conflicting data about the co-existence of other metastases at the time of discovery of TM; Surov and colleagues [19] showed that TM was the unique metastasis in 76% of their patients, while Hegerova et al. [10] documented contrary data, finding that 79% of patients were also affected by metastasis in other organs. These divergent results could be partly explained by the different approaches and extensions to oncological follow-up investigations adopted by the authors.

The vast majority of TMs are metachronous [20], detected after a mean time of 2.3 years in head and neck cancers [10,21], a mean time of 9.4 years in renal cell carcinoma [22], and even after 21 years in intestinal neuroendocrine tumors [23]. In our literature review, we found 38 papers published between 2000 and the end of January 2023 that satisfied our search criteria, consisting of 58 cases of TM that originated from BC [5,9,11,17,18,24,25,26,27,28,29,30,31,32,33,34,35,36,37,38,39,40,41,42,43,44,45,46,47,48,49,50,51,52,53,54,55]. We summarized the cases in Table 2. All the reported patients were female, as expected, and the mean age was 54.2 years. When specified (in forty-three out of the fifty-eight cases), the ductal type resulted in the most frequent histology of breast cancer (28 out of 43 cases, 65%), followed by four cases of lobular type (9.3%); five (11.6%) that were defined as invasive; two (4.6%) that were poorly differentiated; and one (2%) that was defined as “ductal and lobular”, medullary, metaplastic and mucinous. Of the patients, thirty-seven out of fifty-eight (63%) had sites other than the thyroid involved with metastatic disease, especially the lungs, bones and lymph nodes, while eighteen (31%) showed solitary TM and three (6%) had no relevant data recorded. The patient we report was affected by new hepatic and secondary bone disease. The average time of onset from the primary diagnosis of BC was of 70 months, ranging from simultaneous appearance [18,24,25,44] to 180 months [5,46]. Notably, our patient demonstrated TM 18 years (216 months) after the diagnosis of BC, the largest time interval reported to date. The occurrence of TM secondary to BC does not show any peculiar age relation, and of course, has a female predilection. TM can have different modes of presentation. It usually comes to the patient’s and/or the clinician’s attention as a palpable neck mass, which is then confirmed through an ultrasound or CT scan; however, sometimes these thyroid nodules can cause signs and symptoms of dysphagia, hoarseness, dysphonia and pain due to local invasiveness. Our patient developed severe dysphagia that limited her food and liquids intake, with subsequent weight loss. Moreover, her profuse asthenia and weakness were also exacerbated by the severe hypothyroidism and hypoparathyroidism that arose from the substitution of both gland tissues by the TM. In fact, one year before our first visit, the patient had a documented history of normal thyroid and calcium metabolism (Table 1), with no history of hormonal or replacement therapy. Only a total thyroidectomy or eventual ablation with radioactive iodine could cause such a rapid thyroid function deterioration, and neither of these had been performed. Finally, serological and histopathological investigations ruled out autoimmune thyroiditis, which is a leading cause of chronic (not acute) hypothyroidism in our population. All of these elements made our etiopathogenetic hypothesis the most plausible. This is noteworthy, since it made our patient come to our attention and it opened the way to diagnose the secondary malignancy years after the BC treatment. There are no other cases in the literature characterized by a comparable impairment of the thyroid and parathyroid function in a similar clinical context. There was one peculiar presentation that consisted in fever, neck pain and rigidity exacerbated by swallowing in a 37-year-old woman with a three year history of BC, which was initially diagnosed as acute thyroiditis, however the authors did not report the patient’s thyroid function test at the time nor on previous occasions [29].

Ultrasound is the preferred imaging approach to evaluate thyroid diseases, including TM. Previous studies [56,57] agreed that secondary thyroid neoplasms can be classified into two categories, based on ultrasonographic results: (i) diffuse type, appearing as hypoechoic lesions involving the entire gland, and (ii) nodular type, appearing as hypoechoic and hypovascularized nodular lesions. In our case, the TM appeared as bilateral hypoechoic nodules with ill-defined margins and punctate hyperechoic foci, corresponding to microcalcifications, in agreement with what had already been described by other authors in patients whose primary malignancy was BC [39,49]. Nevertheless, we missed the full extent of the disease, which caused the onset of dysphagia and glands deficiencies, underlining the limited reliability of thyroid ultrasound examination due to its rare occurrence in clinical imaging, even among skilled operators. Even the ultrasound risk stratification of malignancy, recently introduced by several scientific societies to assess thyroid nodules, can miss malignant lesions [58].

**Figure 3:**
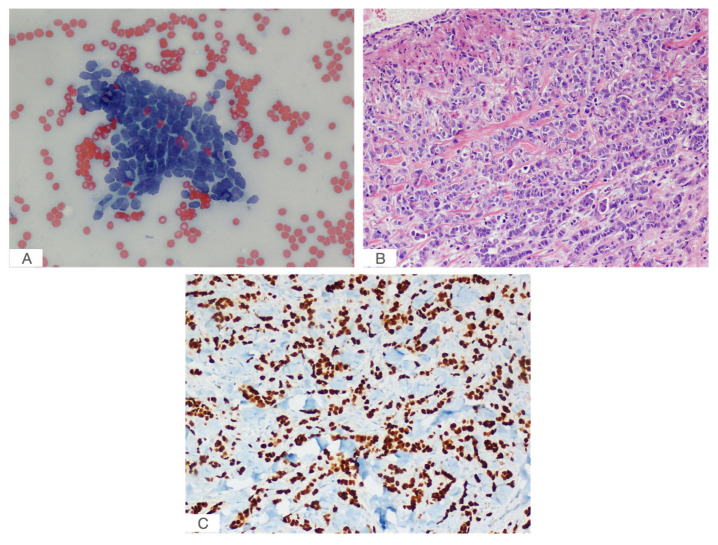
(**A**) Papanicolau-stained FNAC of the right thyroid lobe nodule (40× magnification); (**B**) hematoxylin and eosin (H&E) staining of the right thyroid lobe revealed malignant epithelial cells (20× magnification); (**C**) IHC staining showed that malignant cells in thyroid were positive for GATA3 (20× magnification).

Cytologic evaluation with FNAC is the exam of choice to establish the benign or malignant nature of thyroid lesions. However, less than 0.2% of thyroid FNACs [38,39] reveal a BC metastasis, and it is generally challenging to demonstrate the origin of the primary metastatic cancer [40]. FNACs of thyroid metastatic disease from BC usually consist of malignant epithelial cells with enlarged nuclei and irregular nuclear contours [33], without intranuclear grooves and pseudoinclusions. Sometimes, the cytological samples can even mimic primary TC [38], including C cell hyperplasia and medullary thyroid carcinoma [59]. In our case, the FNAC of the thyroid showed epithelial-like cells with pronounced nuclear atypia (Figure 3A).

In this context, immunohistochemistry (IHC) can support a differential diagnosis between TM and primary TC, which are mostly TG, TTF-1 and PAX8 positive [60], whereas these markers are negative in secondary thyroid malignancy; ER, PR, HER2, GATA3 and GCDFP15 are peculiar of BC-derived tissues [13,61]. Historically, through the identification of hormone and other oncogene receptor status, IHC allowed clinicians to describe several BC subtypes with distinct biological behaviors. This has been translated into a very useful clinical tool and has improved therapies for BC. Furthermore, as highlighted by previous study [62], the receptor status could also be correlated to site-specific metastasis patterns. In fact, beside bone metastasis, which is observed with all subtypes, brain metastasis seems to be more frequent in HR−/HER2+ patients and liver metastasis is more frequent in HER2+ than in HER2− subtypes, while secondary lung involvement is more common in triple negative (TN) tumors [13]. Our case was ER (+), PgR (+) and HER2 (−), and this pattern may be highly adaptive to the thyroid microenvironment in order to initiate organ metastasis; however, further data are needed to establish this relationship.

The presence of secondary thyroid malignancy implies a poor prognosis [62,63], especially if it is synchronous with the primary tumor [6]. Previous experiences documented that after the discovery of TM, the mean survival is three years, or six years if we take into consideration the time of the diagnosis of a primary malignancy [10].

Prospective studies on the impact of surgery for metastatic disease of the thyroid are lacking, although Romero et al. demonstrated that thyroidectomies can ameliorate the prognosis of these patients [64]. Isolated thyroidectomies have a crucial role in limiting the local disease burden in order to alleviate the potential morbidity secondary to the involvement of the upper airway [65]. Of the fifty-eight cases in our review, thirty-six (62%) displayed the adopted management strategy and of these, twenty-one (61%) were treated exclusively with surgery (fifteen (71%) with total or near-total thyroidectomy, six (29%) with partial thyroidectomy), thirteen (36%) with systemic treatments and two (5%) with both. Our patient undoubtedly benefited from the subtotal thyroidectomy, which saw the complete regression of the dysphagia in a short time, and the later systemic therapy that was also implemented.

## 5. Conclusions

BC may metastasize to the thyroid gland. The clinical onset of TM can be heterogeneous or even silent and it is often an incidental finding of investigations undertaken during the follow-up care of cancer patients. We reported a rare case of TM which had originated from BC treated 18 years earlier, which had become clinically evident with severe dysphagia, hypothyroidism and hypoparathyroidism caused by the substitution of the gland tissues by the metastasis. Therefore, it is necessary to remain vigilant on the possible appearance of this type of rare distant disease, even many years after the treatment of the primary cancer. Ultrasound of the thyroid, FNAC, IHC and surgical excision are all valid tools that can help to correctly manage these challenging cases.

## Figures and Tables

**Table 1:** Thyroid function test before, during and after the diagnosis and management of patient TM.

	1 Year before TM Diagnosis	At TM Diagnosis	With L-T4 Therapy *
TSH (mUI/L) [0.4–4]	1.18	27.700	2.08
fT3 (ng/L) [2.70–5.70]	N/A	2.55	2.78
fT4 (ng/dL) [0.70–1.70]	N/A	0.94	1.22

TM—thyroid metastasis; N/A—not available; TSH—thyrotropin; fT3—free triiodothyronine; fT4—free thyroxine; L-T4—levothyroxine; * 100 mcg/day.

**Table 2:** Summary of clinical characteristics of thyroid metastasis from breast cancer reported in the literature since 2000.

Reference	Publication Year	Number of Patients	Sex	Age	BC Histology	Other Metastasis	Time Interval between BC and TM (Months)	Clinical Presentation	TM Management	Follow-Up Since TM Diagnosis (Months)
Bult et al. [26]	2000	1	F	64	Invasive	None	144	Palpable thyroid nodules	CT, RT	10
Chung et al. [27]	2001	6	F	49	-	Lung, bone	-	-	-	-
			F	61	-	Lung	-	-	-	-
			F	51	-	Lung, bone, liver	-	-	-	-
			F	32	-	Lung, liver	-	-	-	-
			F	22	-	Bone, peritoneum	-	-	-	-
			F	33	-	Lung	-	-	-	-
Loo et al. [28]	2003	1	F	52	Ductal	Bone	96	Palpable thyroid nodules	CT	24 alive
Jimenez et al. [29]	2004	1	F	37	-	None	36	Acute Thyroiditis	Total Tx	7 alive
Gong et al. [30]	2005	1	F	57	Metaplastic	None	24	Palpable thyroid nodules, hoarseness	-	-
De Ridder et al. [31]	2003	1	F	-	Lobular	None	-	-	Hemi-Tx	19
Wood et al. [5]	2004	1	F	72	Invasive	None	180	-	Total Tx	36 alive
Kim et al. [9]	2005	5	F	36	Ductal	Lung, LN	18	Palpable thyroid nodules	-	-
			F	34	Ductal	Lung, scalp	25	Multinodular goiter	-	-
			F	44	Ductal	None	37	Palpable thyroid nodules	-	-
			F	55	Ductal	Lung, parotid gland	68	Multinodular goiter	-	-
			F	45	Ductal	Neck LN, lung, bone	85	Palpable thyroid nodules	-	-
Leboeuf et al. [32]	2006	1	F	59	Ductal	Mediastinal, lung, LN, adrenal	168	Palpable thyroid nodules	Total Tx	12
Owens et al. [33]	2005	1	F	64	Invasive	None	60	Neck swelling and pain	CT	-
Peteiro et al. [25]	2005	1	F	42	Ductal	None	0	Palpable thyroid nodules	Hemi-Tx	-
Cichon et al. [34]	2006	1	F	50	-	None	120	Multinodular goiter	Total Tx	24 alive
Skowronska Jozwiak et al. [24]	2010	2	F	49	Lobular	None	0	Palpable thyroid nodules	Total Tx	-
			F	65	-	Lung	48	Palpable thyroid nodules	-	-
Lacka et al. [35]		1	F	54	Ductal and Lobular	Bone, adrenal	168	Multinodular goiter	Total Tx	36 alive
Nguyen et al. [36]	2013	1	F	67	Lobular	None	48	-	-	-
Liu et al. [37]	2014	1	F	47	Ductal	None	24	Palpable thyroid nodules	-	-
Magers et al. [38]	2016	1	F	37	Ductal	Brain, bone	72	-	-	-
Plonczak et al. [11]	2017	1	F	62	Ductal	Lung, bone	144	Neck swelling	Total Tx	14 alive
Zhou et al. [39]	2017	8	F	48	Poorly differentiated	Chest wall	84	-	CT	14 alive
			F	59	Invasive	Chest wall	24	-	CT	5 alive
			F	57	Invasive	Lung, LN	108	-	CT	21 alive
			F	67	Ductal	None	74	-	CT	4 alive
			F	48	Ductal	Lung	120	-	Total Tx	15 alive
			F	52	Ductal	None	6	-	Hemi-Tx	45 alive
			F	69	Poorly differentiated	None	60	-	Total Tx	38 alive
			F	43	Medullary	LN	84	-	CT	30 alive
Pensabeme et al. [40]	2018	1	F	64	Lobular	None	6	Multinodular goiter	Hemi-Tx	32
Durmo et al. [41]	2019	1	F	72	Ductal	-	-	18F-FFDG PET/CT staging	-	-
Zhang et al. [42]	2019	1	F	38	Ductal	LN	24	Palpable thyroid nodules	Total Tx	5 alive
Pakula et al. [43]	2019	1	F	66	-	-	48	Staging	Hemi-Tx	36
Raveendrannair et al. [18]	2019	1	F	36	Ductal	Lung	0	Staging	Total Tx	-
Lee et al. [44]	2020	1	F	64	Ductal	-	0	18F-FDG PET/CT staging	Total Tx	-
Wang et al. [45]	2020	1	F	58	Mucinous	None	156	Neck swelling	Tx	9 alive
Jung et al. [46]	2020	1	F	59	-	LN, bone	180	Neck swelling and hoarseness	CT	39
Mistelou et al. [47]	2019	3	F	62	Ductal	Pleura, chest wall, lung, heart, liver	-	-	-	-
			F	76	Ductal	Pleura, chest wall, bone, lung, adrenal	-	-	-	-
			F	76	Lobular	Chest wall, lung, bone, pleura, liver	-	-	-	-
Wen et al. [48]	2022	1	F	49	Ductal	Neck LN	36	Palpable thyroid nodules	CT	6 alive
Wang et al. [49]	2021	1	F	54	Ductal	Neck LN, chest wall	22	Palpable thyroid nodules, enlarged bilateral neck LN	CT	-
Kiziltan et al. [17]	2021	1	F	70	Ductal	LN, bone,	144	Staging	-	-
Yu et al. [50]	2021	1	F	59	Ductal	LN, chest wall	60	18F-FDG PET/CT staging	Total Tx, CT	12 alive
Celik et al. [51]	2022	1	F	78	Ductal	None, lung, mediastinal LN	0	18F-FDG PET/CT staging	CT	3 alive
Gharib et al. [52]	2022	1	F	48	-	Bone, lung, pleura	0	Staging	Total Tx	-
Yang et al. [53]	2022	1	F	62	Ductal	LN	60	Palpable thyroid nodules	-	-
Hoshi et al. [54]	2022	1	F	58	Ductal	LN	156	Neck swelling	Total Tx	-
Zhang et al. [55]	2022	1	F	46	Ductal	LN, brain	36	Staging	CT	12
Current Study	2023	1	F	72	-	Liver, LN, peritoneum, bone	216	Dysphagia, severe hypothyroidism	Hemi-Tx	15 alive

F—female; LN—lymph node; BC—breast cancer; TM—thyroid metastasis; CT—chemotherapy; RT—radiotherapy; Tx—thyroidectomy; 18F-FDG PET/CT—18F-fluoro-2-deoxyglucose positron emission tomography/computed tomography.

## Data Availability

The authors confirm that the data supporting the findings of this study are available within the article.
